# Assessment of Antibiotic Dispensing Practice in Community Pharmacies of Tehran, for 2 Common Infectious Symptoms, Using Simulated Patient Method

**DOI:** 10.22037/ijpr.2020.112096.13529

**Published:** 2020

**Authors:** Delaram Soltani, Sholeh Ebrahimpour, Seyed Hossein Hajimiri, Aarefeh Jafarzadeh Kohneloo, Zahra Jahangard-Rafsanjani

**Affiliations:** a *Department of Clinical Pharmacy, Pharmaceutical Sciences Branch, Islamic Azad University, Tehran, Iran. *; b *Department of Clinical Pharmacy, Virtual University of Medical Sciences, Tehran, Iran. *; c *Department of Pharmacoeconomics and Pharmaceutical administration, Faculty of Pharmacy, Tehran University of Medical Sciences, Tehran, Iran. *; d *Research Center for Rational Use of Drugs, Tehran University of Medical Sciences, Tehran, Iran. *; e *Department of Epidemiology and Biostatistics, Faculty of Health, Tehran University of Medical Sciences, Tehran, Iran. *; f *Department of Clinical Pharmacy, Faculty of Pharmacy, Tehran University of Medical Sciences, Tehran, Iran.*

**Keywords:** Antibiotic, Community Pharmacy, Simulation, Sore throat and urinary infection

## Abstract

Resistance to antibiotics is a worldwide concern and community pharmacies can play a strategic role in controlling this issue through rationalizing antibiotic consumption. Considering that dispensing any type of antibiotics without a prescription is prohibited according to Iran’s regulations, this study was conducted to quantify the rate of antibiotic dispensing without a prescription by pharmacists in Tehran, Iran. A descriptive cross-sectional study was conducted from September 2016 through May 2017. Two scenarios of common infectious symptoms including sore throat and dysuria were simulated by pharmacy student in three different regions of Tehran. Each scenario was performed in three levels of demand including requesting for any medicine, asking for a stronger medicine, and direct request for an antibiotic. A total of 388 pharmacy visits were acceptable including 195 and 193 pharmacies for dysuria and sore throat, respectively. Antibiotics were provided in 39.9% of dysuria (67.5% in the first level of demand) and in 52.3% of sore throat (49% in the first level of demand) simulations. The time devoted by the pharmacists to each case was less than 60 second in more than 90% of the cases. The completion of the course of antibiotic therapy was emphasized by pharmacists in only 18% of cases in both scenarios. Our findings revealed that antibiotic dispensing without a prescription is a routine practice in community pharmacies in Tehran, Iran. Unfortunately, patient assessment and evaluation of the symptoms are not performed properly by pharmacists as well.

## Introduction

Since their discovery, antibiotics have been one of the most important groups of medicines and have revolutionized human confrontation with infections. Recent medical achievements, such as organ transplantation and cancer chemotherapy are beholden to the discovery of antibiotics. Besides the benefits of these agents in the treatment of infectious diseases, resistance to existing antibiotics has become a global challenge in recent decades. Unfortunately, the rate of resistance to antibiotics has exceeded the rate of developing new antibiotics (1, 2).

 Widespread use of antibiotics leads to microbial resistance and many first-line antibiotics are not effective anymore at usual doses. The results of previous studies suggest that antibiotic resistance leads to prolonged of hospital stay and increases morbidity and mortality in patients with infectious diseases. Moreover, antibiotic resistance imposes a significant financial burden to the population that is exposed (3).

The emergence of antibiotic resistance in Iran has been accelerated by misuse and overuse of antibiotics, which exposes the health care system to serious problems (4). Antibiotic Self-medication for the treatment of minor health problems is highly prevalent in Iran. Results of previous studies indicated respiratory tract symptoms including flu, sore throat, and cough were the most common use of antibiotics as self-medication (5, 6). Antibiotic self-medication is frequently stated as one of the main factors contributing to drug resistance (7). The results of a study conducted in the southeast of Iran in 2010 revealed that one-third of the participants reused antibiotics prescribed once by the physician. Interestingly, most of them did not know that it was a kind of self-medication (8). In another study conducted in community pharmacies in Tabriz, nearly 50% of non-prescription medication requests were for prescription-only medicines such as antibiotics (9). This is while providing antibiotics without a prescription is strictly prohibited by the Iranian Food and Drug Organization. However, this law is not properly followed by pharmacists, and selling medicine is usually done at the patient’s request. Of note, the majority of the pharmacy income in Iran depends on selling medications rather than providing patients with counseling services. On the other hand, the dispensing fee is not adequate in Iran and is not covered by most of the insurers. These factors can partly justify the interest of the pharmacists in providing medicines without a prescription in Iran (10). 

The current study was conducted to evaluate the behavior of pharmacists when asked for advice for two common infectious symptoms in Tehran community pharmacies. In order to increase the conformity of the results obtained from the study with real conditions in the society, the method of the simulated patient method was applied. A simulated patient is a person who is well trained to display a predetermined scenario. By conducting this method, real practice of subjects could be evaluated. The use of the simulated patient method for evaluating the pharmacists’ behaviors is widely performed in the context of pharmacy practice (11). However, this method is less considered in this area of practice in Iran. To the best of our knowledge, the only relevant published investigation in Iran is limited to the study conducted by Sima *et al.*, in 1976. In the mentioned study, the simulated patient method was used to evaluate drug dispensing without prescription on sore throat scenario in only 30 pharmacies (12). 

## Experimental

A descriptive cross-sectional study was conducted from September 2016 through May 2017, in Tehran, Iran. Four hundred and five out of 1442 community pharmacies across five geographic regions of Tehran were approached. A sample size of 405 was calculated based on an estimated correct practice about 50%, assuming. The estimated rate of correct practice was based on a pilot study which included 50 community pharmacies. In the main study, the number of included pharmacies in each region was proportional to the total number of pharmacies in that area. Selection of pharmacies was done using convenient sampling method.

The pilot study was conducted to find any problem or error in the role-playing practice and to revise the questionnaire before starting the main study. The design of the questionnaire was based on seven items to completely cover the answers of each pharmacist. The questionnaire was filled out immediately after visiting the pharmacy. Different parts of the questionnaire are detailed in Table 1.

Two scenarios, each in three levels of demand, were designed to assess non-prescription antibiotic dispensing for sore throat and dysuria. A pharmacy student role played both scenarios. The first level of demand consisted of asking for a medicine to treat a sore throat or burning sensation during urination. If an antibiotic was not dispensed during the first demand, the simulated patient asked a stronger medicine during the second level. In the case of referring to a physician, the simulated patient insisted on buying medicine from the pharmacy. Direct asking for an antibiotic was the third level of demand which was carried out when no antibiotic was dispensed in the second level. Only one scenario was simulated in each specific pharmacy. In each simulation, it was assured that the consulting person was the pharmacist in charge, not the pharmacy staff by checking the identification tag or asking to talk with the pharmacist. The age of pharmacist was estimated based on the appearance. In all cases, the offered medicine by the pharmacist was purchased by the simulated patient. Time allocated to the patient by the pharmacist was reported based on the simulated patient estimation.

Additional information was given only when the pharmacist asked about it. The details of the two scenarios, predicted questions, and the goal of each one are shown in Table 2. These two scenarios were designed based on the common symptoms and criteria in the diagnosis of bacterial pharyngitis and urinary tract infection (13, 14). 

Ethical clearance was obtained from the Ethics Committee of Tehran University of Medical Sciences (Ethics Code: IR.TUMS.VCR.REC.1395.965).

The results are reported as numbers and frequencies. Chi-square test was used to compare the frequency of antibiotic dispensing and frequency of referral to a physician between the two scenarios and evaluate the association between geographic regions and the frequency of antibiotic dispensing. In all analyses, *p*-values < c 0.05 were considered as statistically significant. Data analysis was performed using SPSS statistics software version 21.0 (IBM Corp. Armonk, NY, USA).

## Results

A total of 405 pharmacies were approached in this study. Seventeen pharmacies were excluded as the simulated patient was recognized by the pharmacist or staff. One hundred and ninety-five pharmacies were evaluated using the sore throat scenario and 193 pharmacies using the dysuria scenario. 

Antibiotics were sold without a prescription in 102 (52.3%) pharmacies where the sore throat was simulated and in 77 (39.9%) pharmacies where dysuria was simulated. Compared to the sore throat scenario, the frequency of antibiotic dispensing by the pharmacists was significantly lower in the dysuria scenario (*p* = 0.008). The distribution of approached pharmacists based on gender was 189 (48.7%) females and 199 (51.3%) males. The estimated age of pharmacists based on appearance was ≥ 30 years in 101 (26%) cases and < 30 years in 287 (74%) pharmacies. Considering all of the 388 pharmacies, the rate of antibiotic dispensing was not significantly different according to gender (49.8% for males *vs* 42.8% for females, *p* = 0.1), but it was considerably different according to two age categories (70.3% in ≥ 30 years *vs* 38% in < 30 years, *p* < 0.0001).

Forty-four of 195 (22.8%) and 122 of 193 (62.8%) cases were referred to a physician in sore throat and dysuria scenarios, respectively. Our findings revealed that the rate of referral to a physician was considerably higher in the dysuria scenario compared to sore throat (62.8% *vs* 22.8%; *p* = 0.001).

The rate of dispensing antibiotics at the first level of demand was similar in both scenarios (Table 3). At the second level of demand, the proportion of antibiotic dispensing was significantly higher in the sore throat scenario compared to dysuria (18.4% *vs* 7.3%; respectively). The comparison of antibiotic dispensing in three different levels of demand between the two scenarios is shown in (Table 3).

The rate of antibiotic dispensing in the five geographical regions was significantly different in the sore throat scenario indicating an increasing trend from the northern to southern regions (35.7% to 72.4%; *p* = 0.030). The community pharmacies in each region and the frequency of antibiotic dispensing are shown in Table 4.

Among 102 pharmacies dispensed an antibiotic for the treatment of sore throat, amoxicillin 500 mg (68 pharmacies; 66.7%) was the most frequently sold antibiotic followed by azithromycin 250 mg (17 pharmacies; 16.7%) and cefixime 400 mg (11 pharmacies; 10.8%). Of 77 pharmacies that sold an antibiotic for dysuria, ciproﬂoxacin 500 mg (59 pharmacies; 76.6%) and co-trimoxazole 400/80 mg (8 pharmacies; 10.4) were the most frequently sold antibiotics and nitrofurantoin was only suggested in one case. Clindamycin vaginal cream 1% and fluconazole capsule 150 mg were each dispensed in one case without any question about the history of sexually transmitted diseases or vaginal discharge.

In the sore throat scenario, 122 (62.6%) pharmacies committed to dispensing medicines including antibiotics and non-prescription products. In 102 (52.3%) cases, antibiotics and non-prescription medicines were dispensed simultaneously. Cold candy (37 pharmacies; 30.3%), diphenhydramine elixir (35 pharmacies; 28.7%), and herbal products (20 pharmacies; 16.4%) were the most common non-antibiotic prescription medicines recommended by the pharmacists in sore throat scenario. 

Taking phenazopyridine or drinking lots of water (24 pharmacies, 20.7%) were recommended for the alleviation of dysuria by pharmacists who did not dispense antibiotics and did not refer the simulated patient to a physician. In addition, 92 pharmacies (79.3%) did not sell any drugs. 

In more than 90% of the cases (374 0f 388), the time devoted to counseling by the pharmacist was less than 60 sec. It also should be noted that only 26 (32.5%) pharmacists in the dysuria and 64 (62.7%) pharmacists in the sore throat scenario that sold an antibiotic asked about other symptoms, which were significantly higher in the sore throat scenario (*p* = 0.001). The onset of symptoms, the other therapeutic measures, taken to eliminate the symptoms, the other drugs used by the patient, the history of drug allergy, and the pregnancy/breastfeeding were only asked in less than 5% of the cases. In dysuria, the simulations for which antibiotics were dispensed, supplementary questions such as the presence of fever, as well as hematuria and back pain for distinguishing complicated from uncomplicated UTI were not asked. The dose of antibiotics was mentioned in 69 (89.6%) cases of dysuria and 70 (68.6%) cases of sore throat. Moreover, the duration of treatment was only mentioned by 14 (18.2%) and 19 (18.6%) cases for dysuria and sore throat, respectively. 

Concerns about the rate of antibiotic resistance were stated in only 4 cases (1.9%) in whom the pharmacist refused to dispense antibiotics.

## Discussion

The most remarkable result of the current study was that nearly half of the pharmacists dispensed antibiotic without prescription in both scenarios (179 out of 388 cases). The findings showed that antibiotics were dispensed and could be easily purchased without a prescription in community pharmacies of Iran, especially for treatment of suspected bacterial symptoms.

The simulated patient strategy used in the current study can closely mimic what happens in the real-world situation. Therefore, the results may reliably be extrapolated to the real setting.

Similar studies were also conducted in other countries when the global attention about antibiotic resistance had been intensified (15-18). Despite the different rates of dispensing antibiotics without a prescription, the results were similar in these studies, suggesting irrational antibiotic use in different clinical cases in community pharmacies. It occurs even in the developed countries, as antibiotics were dispensed in about 45% of community pharmacies in Catalonia when they were evaluated by sore throat, dysuria, and acute bronchitis scenarios (17). 

Our finding revealed that the rate of antibiotic dispensing was not significantly different between the male and female pharmacists. The results of the current study did not support a previous investigation conducted by Gastelurrutia *et al*. That showed that male staff were more likely to provide antibiotics without a prescription (19). 

It is interesting to note that the rate of dispensing antibiotic in the current study was affected by the pharmacist’s age. Younger pharmacists were less interested in providing antibiotics. Since the younger pharmacists graduated recently, they may be more concerned about the unintended consequences of the irrational use of antibiotics.

We found that pharmacists were less likely to dispense antibiotics in a case of dysuria compared to sore throat. This finding was not in line with the results of a study by Lior *et al.,* which showed the rate of antibiotic dispensing by pharmacists was higher in the dysuria scenario compared to the sore throat scenario (34.8% *vs* 79.7%) (17). Although Lior and collogues did not provide any explanation, a possible reason might be that in these cases, sore throat was considered as a sign of viral infection, and as a result, the pharmacists had a lower tendency to dispense antibiotics. 

The results of the current study revealed that about half of antibiotic dispensing for sore throat and two-third of dispensing for dysuria happened in the first level of demand without any insistence. Even a higher rate of antibiotic dispensing (95%) in the absence of any patient demand, was reported in the study conducted in Saudi Arabia (20). Moreover, our findings showed at the second level of demand, more antibiotics were dispensed in the sore throat scenario in comparison to dysuria cases. The same results were observed with less difference in the third level of the demand. On the other hand, a substantially higher rate of referral to a physician was observed in the dysuria scenario, compared to the sore throat (*p* = 0.001). Finally, the results of the current study indicated less antibiotic dispensing in the dysuria scenario in parallel with further referral to a physician. These results may be explained to some extent by the complexity of underlying causes of dysuria, leading to the lower tendency of pharmacists to antibiotic dispensing and raise the rate of referral to a physician. 

The findings of the present study suggest that the rate of antibiotic dispensing in the sore throat scenario was considerably affected by geographical region. Perhaps socio-economic differences were involved in its creation. The lower socio-economic level of the southern regions (21). led to the more antibiotic dispensing and a lower rate of accepting remedies other than antibiotics in comparison with other regions. 

The influence of socioeconomic status, culture, and education on the pattern of antibiotic use without a prescription has addressed in various studies (22, 23). The result of a study conducted in Spain to evaluate the social determinants of antibiotic use revealed a strong association between lower educational levels and a higher rate of antibiotics consumption (22). Another study was conducted by Deschepper *et al.,* to assess the effect of culture on the pattern of antibiotic use in a city in Belgium and a city in the Netherlands. The result of this study showed that in the Netherlands, upper respiratory symptoms were often considered as a result of the common cold or flu. However, in Belgium, these symptoms were mostly regarded as bronchitis and antibiotics were used to treat them. (23). 

In line with similar studies, Amoxicillin was the most common dispensed antibiotic when a sore throat scenario simulated (24). Surprisingly, amoxicillin was considered as a non-prescription medicine in many cases without any referring to a physician, although for the rest of the dispensed antibiotics, referring to a physician occurred concurrently. In the case of dysuria, ciprofloxacin ranked the first among the dispensed antibiotics. Interestingly, this vital antibiotic was administered without appropriate evaluation, which leads to the overuse of antibiotics and increases the risk of microbial resistance. 

In more than 90% of the cases, the estimated allocated time to each case by the pharmacist was less than 60 sec and antibiotics were dispensed without sufficient initial assessment. Patient assessment did not follow a correct pattern, so questions about the history of allergy, pregnancy status, onset of symptoms, and patient’s other medications were asked in only less than 5% of the cases. On the other hand, if the simulated patient was properly evaluated, all dysuria cases should have been referred based on the scenario. This was in contrast to the sore throat scenario in which there was no need for referring the patient to a physician and symptom alleviation could be obtained by offering non-prescription medicines. Lack of a systematic approach to the patient to ascertain the need for referral was observed in this study. 

In addition, the type of the provided information for dispensed antibiotics mainly focused on dosing while parameters like the duration and directions for use, precautions, and contraindications and adverse reactions were poorly addressed. Moreover, in both scenarios, completion of the treatment course was emphasized by pharmacists in only 18% of the cases for those who received antibiotics. Similarly, Surur *et al.,* found that important information such as food-drug interactions, management of missed doses, and contraindications were ignored in more than 50% of the cases (25). This can exacerbate the rising rate of antimicrobial resistance. 

Pharmacists participating in the face-to-face interview in Saudi Arabia stated the ease of access to pharmacists compared to other health care provider, patients’ trust in pharmacists, the lack of strong regulatory enforcement, customer pressure, and concerns about the financial survival of the pharmacy are the most common reasons for dispensing antibiotics without prescription through the pharmacies (26). On the other hand, suboptimal counselling practice was found in our study. Similar results were reported in the different studies. A high volume of prescriptions, time constraints, the unwillingness of the patients to receive supplementary information from the pharmacist, and not allocating fees to the patient counseling services, have been cited by pharmacists in several community pharmacists-based surveys as the deterrent in providing counseling service. (27). 

According to the results of this study, it can be claimed that pharmacists are not sufficiently aware of their vital role in reducing the rate of antibiotic resistance by limiting unnecessary uses of antibiotics. It seemed that they are desensitized to dispensing antibiotics and its correlation with increasing the rate of antibiotic resistance. 

It might be possible to perform knowledge, attitude, and practice studies in future investigations to determine current pitfalls in non-prescription antibiotic dispensing by pharmacists.

The results of the current study showed that antibiotics were dispensed easily without a prescription in the community pharmacies in Tehran, capital city of Iran. Reinforcement of existing regulations regarding antibiotics dispensing by regulatory organizations seems necessary in the current situation. It seems that pharmacists need complementary educational courses to execute their crucial role in rationalization of antibiotic use. 

**Table 1:** Details of scenario 1 and 2 in which the simulated patient requested a medicine to treat sore throat or dysuria

**Scenario 1**	**Goal of scenario 1**
The medicine was requested for simulated patient’s sister (Age: 20) who had moderate to severe sore throat for 2 days.	The goal of the scenario was to simulate a viral sore throat and pharmacists were expected to dispense OTC medicines to alleviate symptoms as there was no indication for antibiotic dispensing.
(score of 5 based on a 0-10 scale)	
Associated symptoms if they were asked and the answers of the simulated patient:	
Open-ended question: runny nose	
Close-ended questions:	
Cough, sneezing, itchy throat, difficulty swallowing: Yes	
Fever, tonsillar exudates, inflation in lymphatic nodes, neck stiffness, tender anterior cervical adenopathy: No	
Present medications and/or illness: None	
Smoking habit: Negative	
Any action was taken: No	
Pregnancy: No	
Allergy to any medicine: No	
**Scenario 2**	**Goal of scenario 2**
The medicine was requested for the simulated patient herself (Age: 24, married) who had a burning sensation on urination (dysuria) for one day.	The goal of the scenario was to show an uncomplicated UTI requiring more investigation. Pharmacists were expected to refer the simulated patient to a physician.
Associated symptoms if they were asked and the answers of the simulated patient:	
Open-ended question: frequent urination	
Close-ended questions:	
Vaginal discharge, urgency: Yes	
Back pain, fever/chills, hematuria, abdominal pain, change in urine color or odor: No	
Present medication and/or illness: None	
History of UTI: Negative	
Any action was taken: No	
Pregnancy: No	
Allergy to any medicine: No	

**Table 2:** Details of the questionnaire completed by the simulated patient

**Primary information**	**Date, scenario (sore throat/UTI)**
Patient assessment	Who is the medicine asked for? Age, associated symptoms, duration, previous treatments/actions, any other medications, allergies, pregnancy were evaluated by the pharmacist?
Providing information about dispensed medication	Information about indication, doses, advices on completing the duration of treatment, adverse effects, storage condition, precautions were provided by pharmacist?
Result of scenario	Antibiotic dispensed? (Yes/No); If yes: in which level of demand? (First, second or third level) Which antibiotic dispensed? Did patient referred to a physician (Yes/No)
Pharmacist information	Sex Apparent age (under 30, 30 or more, not recognizable)
Pharmacy information	Region
Other information	Time of visiting pharmacy, waiting time in pharmacy, time dedicated to simulated patient, number of patients in pharmacy

**Table 3:** Antibiotic dispensing rates based on level of demand in both scenarios in 388 pharmacies in Tehran

**Level of demand**	**Sore Throat (n = 195)**	**Dysuria (n = 193)**	***p*** **-value**
Level 1- Can you give me a medicine? n (%)	50 (25.6)	52 (26.9)	*p* = 0.018
Level 2- Can you give me a stronger medicine? n (%)	36 (18.4)	14 (7.3)	
Level 3- Can you give me an antibiotic? n (%)	16 (8.2)	11 (5.7)	
Total, n (%)	102 (52.3)	77 (39.9)	*p* = 0.008

**Table 4:** Antibiotic dispensing rates in both scenarios based on different geographic regions in 388 pharmacies in Tehran

**Region**	**Sore Throat (n = 195)**	**Dysuria (n = 193)**
North	15 / 42 (35.7%)	16 / 41 (39.0%)
Center	24 / 43 (55.8%)	14/43 (32.6%)
East	20/42 (47.6%)	17/44 (38.6%)
West	22/37 (59.5%)	16/37 (43.2%)
South	21 / 29 (72.4%)	14 / 30 (46.7%)
*p*-value	*p* = 0.031	*p *= 0.78

## Authors’ Contribution 

Sholeh Ebrahimpour contributed in literature search, data analysis, and manuscript revision. Delaram Soltani, Seyed, Hossein Hajimiri, and Zahra Jahangard-Rafsanjani were involved in study design, data collection, and writing manuscript. Aarefeh Jafarzadeh Kohneloo participated in designing and data analysis. All authors read and approved the manuscript.

## References

[1] Laxminarayan R, Duse A, Wattal C, Zaidi AK, Wertheim HF, Sumpradit N, Vlieghe E, Hara GL, Gould IM, Goossens H, Greko C, So AD, Bigdeli M, Tomson G, Woodhouse W, Ombaka E, Peralta AQ, Qamar FN, Mir F, Kariuki S, Bhutta ZA, Coates A, Bergstrom R, Wright G (2013). Antibiotic resistance- the need for global solutions. Lancet Infect Dis..

[2] World Health Organization (2015). Global action plan on antimicrobial resistance.

[3] Cassini A, Högberg LD, Plachouras D, Quattrocchi A, Hoxha A, Simonsen GS, Colomb-Cotinat M, Kretzschmar ME, Devleesschauwer B, Cecchini M, Ouakrim DA, Oliveira TC, Struelens MJ, Suetens C, Monnet DL (2019). Burden of AMR Collaborative Group. Attributable deaths and disability-adjusted life-years caused by infections with antibiotic-resistant bacteria in the EU and the European Economic Area in 2015: a population-level modelling analysis. Lancet Infect Dis..

[4] Malekolkottab M, Shojaei L, Khalili H, Doomanlou M (2017). Clinical Response and Outcome in Patients with Multidrug Resistant Gram-negative Infections. J. Res. Pharm. Pract..

[5] Jalilian F, Hazavehei SM, Vahidinia AA, Jalilian M, Moghimbeigi A (2013). Prevalence and related factors for choosing self-medication among pharmacies visitors based on health belief model in Hamadan Province, west of Iran. J. Res. Health Sci..

[6] Jafari F, Khatony A, Rahmani E (2015). Prevalence of self-medication among the elderly in Kermanshah-Iran. Glob. J. Health Sci..

[7] Ateshim Y, Bereket B, Major F, Emun Y, Woldai B, Pasha I (2019). Prevalence of self-medication with antibiotics and associated factors in the community of Asmara, Eritrea: a descriptive cross sectional survey. BMC Public Health.

[8] Foroutan B, Foroutan R (2014). Household storage of medicines and self-medication practices in south-east Islamic Republic of Iran. East Mediterr. Health J..

[9] Sahebi L, Vahidi RG (2009). Self-medication and storage of drugs at home among the clients of drugstores in Tabriz. Curr. Drug Saf..

[10] Hashemi-Meshkini A, Keshavarz K, Nikfar S, Vazirian I, Kebriaeezadeh A (2013). Pharmacists remuneration models in iran and selected countries: a comparative study. Iran. J. Pharm. Res..

[11] Watson MC, Norris P, Granas AG (2006). A systematic review of the use of simulated patients and pharmacy practice research. Int. J. Pharm. Pract..

[12] Amidi S, Ajamee G, Sadeghi HR, Yourshalmi P, Gharehjeh AM (1978). Dispensing drugs without prescription and treating patients by pharmacy attendants in Shiraz, Iran. Am. J. Public Health..

[13] Pelucchi C, Grigoryan L, Galeone C, Esposito S, Huovinen P, Little P, Verheij T (2012). Guideline for the management of acute sore throat ESCMID Sore Throat Guideline Group. Clin. Microbiol. Infect..

[14] Gupta K, Hooton TM, Naber KG, Wullt B, Colgan R, Miller LG, Moran GJ, Nicolle LE, Raz R, Schaeffer AJ, Soper DE (2011). International clinical practice guidelines for the treatment of acute uncomplicated cystitis and pyelonephritis in women: A 2010 update by the Infectious Diseases Society of America and the European Society for Microbiology and Infectious Diseases. Clin. Infect Dis..

[15] Shet A, Sundaresan S, Forsberg BC (2015). Pharmacy-based dispensing of antimicrobial agents without prescription in India: appropriateness and cost burden in the private sector. Antimicrob Resist Infect Control..

[16] Almaaytah A, Mukattash TL, Hajaj J (2015). Dispensing of non-prescribed antibiotics in Jordan. Patient Prefer Adherence.

[17] Llor C, Cots JM (2009). The sale of antibiotics without prescription in pharmacies in Catalonia, Spain. Clin. Infect Dis..

[18] Jamshed S, Padzil F, Shamsudin S, Halimah Bux S, Jamaluddin A, Bhagavathula A, Azhar S, Azmi Hassali M (2018). Antibiotic Stewardship in Community Pharmacies: A Scoping Review. Pharmacy (Basel)..

[19] Gastelurrutia MA, Larranaga B, Garay A, Echeveste Fde A, Fernandez-Llimos F (2013). Impact of a program to reduce the dispensing of antibiotics without a prescription in Spain. Pharm. Pract..

[20] Bin Abdulhak AA, Altannir MA, Almansor MA, Almohaya MS, Onazi AS, Marei MA, Aldossary OF, Obeidat SA, Obeidat MA, Riaz MS, Tleyjeh IM (2011). Non prescribed sale of antibiotics in Riyadh, Saudi Arabia: A Cross Sectional Study. BMC Public Health..

[21] Sadeghi R, Zanjari N (2017). The Inequality of Development in the 22 Districts of Tehran Metropolis. J. Social Welfare..

[22] Garcia-Rey C, Fenoll A, Aguilar L, Casal J (2004). Effect of social and climatological factors on antimicrobial use and Streptococcus pneumoniae resistance in different provinces in Spain. J. Antimicrob. Chemother..

[23] Deschepper R, Vander Stichele RH, Haaijer-Ruskamp FM (2002). Cross-cultural differences in lay attitudes and utilisation of antibiotics in a Belgian and a Dutch city. Patient Educ. Couns..

[24] Khalifeh MM, Moore ND, Salameh PR (2017). Self-medication misuse in the Middle East: a systematic literature review. Pharmacol. Res. Perspect..

[25] Surur AS, Getachew E, Teressa E, Hailemeskel B, Getaw NS, Erku DA (2017). Self-reported and actual involvement of community pharmacists in patient counseling: a cross-sectional and simulated patient study in Gondar, Ethiopia. Pharm. Pract..

[26] Alhomoud F, Almahasnah R, Alhomoud FK (2018). “You could lose when you misuse” - factors affecting over-the-counter sale of antibiotics in community pharmacies in Saudi Arabia: a qualitative study. BMC Health Serv. Res..

[27] Abdelaziz AI, Tawfik AG, Rabie KA, Omran M, Hussein M, Abou-Ali A (2019). Quality of Community Pharmacy Practice in Antibiotic Self-Medication Encounters: A Simulated Patient Study in Upper Egypt. Antibiotics (Basel).

